# Vegetable oils as carbon and energy source for *Aureobasidium melanogenum* in batch cultivation

**DOI:** 10.1002/mbo3.764

**Published:** 2018-12-04

**Authors:** Elke J. van Nieuwenhuijzen, Michael F. Sailer, Edwin R. van den Heuvel, Stephanie Rensink, Olaf C. G. Adan, Robert A. Samson

**Affiliations:** ^1^ Westerdijk Fungal Biodiversity Institute Utrecht The Netherlands; ^2^ Saxion University of Applied Sciences Enschede The Netherlands; ^3^ Xylotrade BV Goor The Netherlands; ^4^ Department of Mathematics and Computer Science University of Technology Eindhoven Eindhoven The Netherlands; ^5^ Department of Applied Physics University of Technology Eindhoven Eindhoven The Netherlands

**Keywords:** *Aureobasidium melanogenum*, batch cultivation, biofinish, carbon source, energy source, linseed oil

## Abstract

Dark homogenous fungal‐based layers called biofinishes and vegetable oils are key ingredients of an innovative wood protecting system. The aim of this study was to determine which of the vegetable oils that have been used to generate biofinishes on wood will provide carbon and energy for the biofinish‐inhabiting fungus *Aureobasidium melanogenum*, and to determine the effect of the oil type and the amount of oil on the cell yield. *Aureobasidium melanogenum* was cultivated in shake flasks with different types and amounts of carbon‐based nutrients. Oil‐related total cell and colony‐forming unit growth were demonstrated in suspensions with initially 1% raw linseed, stand linseed, and olive oil. Oil‐related cell growth was also demonstrated with raw linseed oil, using an initial amount of 0.02% and an oil addition during cultivation. Nile red staining showed the accumulation of fatty acids inside cells grown in the presence of oil. In conclusion, each tested vegetable oil was used as carbon and energy source by *A. melanogenum*. The results indicated that stand linseed oil provides less carbon and energy than olive and raw linseed oil. This research is a fundamental step in unraveling the effects of vegetable oils on biofinish formation.

## 1 INTRODUCTION

Timber situated outdoors and above ground is susceptible to discoloration caused by dark wood staining fungi. Dark staining of untreated wood is common, but also of coated and/or modified wood (De Windt, Bulcke, Wuijtens, Coppens, & Acker, 2014; Gobakken & Westin, 2008; Gobakken, Bardage, & Long, 2013; Metsä‐Kortelainen, Paajanen, & Viitanen, 2011). The use of raw linseed oil and olive oil as a wood impregnating agent has been identified to stimulate the amount of dark stains on the wood surface (Van Nieuwenhuijzen et al., 2015; Van Nieuwenhuijzen, Gobakken, Sailer, Samson, & Adan, 2016). Remarkably, some wood species impregnated with these oils have shown to form a beneficial homogenous kind of fungal‐based staining called biofinish (Sailer, Nieuwenhuijzen, & Knol, 2010; Van Nieuwenhuijzen et al., 2015). However, after outdoor exposure of wood samples impregnated with stand linseed oil and untreated wood, none of the stained wood surfaces could meet the degree of surface coverage and pigmentation criteria of a biofinish. Currently, natural biofinish formation is exploited to manufacture and apply a sustainable wood protection system with decorative and self‐repairing abilities (Xyhlo Biofinish, 2018).

Till now, the growth mechanisms of dark wood staining fungi on the surface of oil‐treated wood are poorly understood. Recent studies show the common presence of *Aureobasidium melanogenum*, formerly known as *Aureobasidium pullulans* variety *melanogenum* (Gostinčar et al., 2014), in natural biofinishes of wood (Van Nieuwenhuijzen, Houbraken, Meijer, Adan, & Samson, 2016). Also the impact of other fungal species, such as *Superstratomyces*, has to be considered (Van Nieuwenhuijzen et al., 2017; Van Nieuwenhuijzen, Houbraken, et al., 2016; Van Nieuwenhuijzen, Miadlikowska, et al., 2016). Although many parameters define the growth conditions on the surface of a material outdoors, for example, rain, air temperature, or solar radiation, one of the controllable parameters is the type of vegetable oil used for the wood impregnation. The presences of oil on the wood surface might have an effect on (a) the spore adhesion of dark staining fungi; (b) the local moisture content at the substrate surface; (c) loosening of dark stained wood fibers; (d) attraction of other organisms, which enable dark mold growth; and (e) the availability of nutrients for wood staining fungi (Van Nieuwenhuijzen et al., 2015). These potential effects have not received much attention. This paper will target the latter aspect.

The use of vegetable oils by fungi, including *A. melanogenum* (Leelaruji, Piamtongkam, Chulalaksananukul, & Chulalaksananukul, 2013), is frequently studied in the light of lipase production (Açikel, Erşan, & Açikel, 2011; Fadiloğlu & Erkmen, 2002; Najjar, Robert, Guérin, Violet‐Asther, & Carrière, 2011). Generally, lipases are described as enzymes that catalyze the hydrolysis of triglycerides to glycerol and free fatty acids at an oil‐water interface (Kudanga, Mwenje, Mandivenga, & Read, 2007; Treichel, Oliveira, Mazutti, Luccio, & Oliveira, 2010). In some studies, a vegetable oil was used as a sole carbon‐based nutrient instead of a commonly used monomeric sugar and biomass growth in time was measured (Del Rio, Serra, Valero, Poch, & Sola, 1990; Molokwu & Okpokwasili, 1997). Several tested vegetable oils and fungal species showed fungal growth. For example, the use of olive oil resulted in growth of *Candida rugosa*,* Malassezia* spp., *Yarrowia lipolytica*,* Aspergillus*, and *Penicillium* (Del Rio et al., 1990; Papanikolaou & Aggelis, 2011; Shibata et al., 2006; Tan & Gill, 1984). In the case of the yeast *Saccharomyces cerevisiae*, contradicting results have been found on its ability to grow on lipid substrates (Darvishi, 2012; Shirazi, Rahman, & Rahman, 1998). In the study of Peeters, Huinink, Voogt, and Adan (2018), colony growth of *A. melanogenum* was demonstrated on layers made of a raw linseed and olive oil. Thus far, it is unknown if *A. melanogenum* can use all types of linseed and olive oil as carbon and energy source for growth.

The linseed and olive oils used to explore natural biofinish formation are complex substrates. Raw linseed oil and olive oil mainly contain triglycerides, but also contain small quantities of mono‐/diglycerides, phosphatides, triterpenes, fat‐soluble vitamins, carotenes, and chlorophylls (Karleskind, 1996). The triglycerides are derived from glycerol (C3 alcohol with three hydroxyl groups) and C16 and/or C18 fatty acids. The fatty acid composition of vegetable oils varies, especially between oil types (Karleskind, 1996; Schuster, 1992). Vegetable oils, including linseed and olive oil, are prone to autoxidation and photo‐oxidation (Juita, Dlugogorski, Kennedy, & Mackie, 2012; Psomiadou & Tsimidou, 2002a, 2002b). Raw linseed oil is highly reactive due to the high number of conjugated polyunsaturated fatty acids, including linoleic acid (Stenberg, Svensson, & Johansson, 2005). Subsequent to oxidation processes the amount of double bonds in the oil will decrease, while cross‐links are formed between fatty acids of different triglycerides (Sailer, 2001; Van den Berg, 2002). Due to this process, a solid film of linseed oil can be formed in the presence of air (Lazzari & Chiantore, 1999). Olive oil mainly contains oleic acid and does not form a solid or highly viscous material upon exposure to air (Rheineck & Austin, 1968). Stand linseed oil is made of raw linseed oil. It is produced by heating raw linseed oil to about 300°C for a few days, generally in the absence of oxygen (Juita et al., 2012; Van den Berg, 2002). The chemical reactions during this process include cross‐linking of triglycerides and isomerization of double bonds (Colombini, Modugno, & Ribechini, 2009; Van den Berg, 2002; Zovi, Lecamp, Loutelier‐Bourhis, Lange, & Bunel, 2011). The final chemical composition of stand linseed oil is hard to measure. To which extent stand linseed oil, but also raw linseed oil and olive oil may be used as carbon and energy source by *A. melanogenum* is hard to predict.

The first aim of the study is to determine which of the vegetable oils that have been used to generate natural biofinishes provide carbon and energy for the growth of the biofinish‐inhabiting *A. melanogenum*. The second aim is to determine the effect of the oil type and the amount of oil on the cell yield of *A. melanogenum* when cultivated with a vegetable oil as a sole carbon‐based nutrient. Raw linseed oil, olive oil, stand linseed oil, and the commonly used nutrient glucose were tested as sole carbon‐based nutrient in a shake flask test setup. Different amounts of carbon‐based nutrients were tested, including second oil additions during incubation, in order to gain insight in oil‐dependent growth. This way, a start was made to understand the effects of the use of different vegetable oils on natural biofinish formation of oil‐treated wood.

## 2 MATERIAL AND METHODS

### 2.1 *Aureobasidium* inoculum

The *A. melanogenum* isolate CBS 140241, obtained from the Westerdijk Fungal Biodiversity Centre, was used for all growth experiments. This isolate originates from oil‐treated wood in the initial stage of biofinish formation (Van Nieuwenhuijzen, Houbraken, et al., 2016). An *Aureobasidium* inoculum was made with young hyaline yeast‐like cells. At first, biomass was obtained of the edge of an 8‐ to 18‐day‐old *Aureobasidium* colony on dichloran 18% glycerol agar and combined in a shake flask with minimal media (MM), including NaNO_3_, KH_2_PO_4_, KCl, MgSO_4_, and trace elements as designed by De Vries et al. (2004), and with 2% glucose (d(+) glucose anhydrous, Merck KGaA). After 24 hr of rotary shaking at 200 rpm at 25°C, the suspension was washed twice and resuspended in 2% glucose MM. After another 24 hr of incubation, the *Aureobasidium* biomass was washed, suspended in MM (so without any carbon), and ready for use.

### 2.2 Growth media

Each suspension contained 98–99 v/v % MM and 1 v/v % *Aureobasidium* inoculum. The total cell concentration in a suspension at the start of cultivation was between 5 × 10^5^ and 10 × 10^5^ cells/ml. The type and amount of the carbon‐based nutrient used varied (Table 1). The different carbon‐based nutrients tested were as follows: raw linseed oil (Vereenigde Oliefabrieken; iodine value 183 and 0.81% free fatty acids), stand linseed oil (Vliegenthart, viscosity P45), olive oil (Carbonel, extra vierge, iodine value 82 and 0.34% free fatty acids), and d (+) glucose (Merck, anhydrous for biochemistry). Most tested media contained the concentration of 1 w/v % (10 g/L), further referred to in this paper as 1% (Table 1). In addition, glucose was used with an initial concentration of 0.2 w/v % (2 g/L) and raw linseed oil with an initial concentration of 0.02 w/v %, further referred to in this paper as 0.2% and 0.02%, respectively. Suspensions with initially 1% vegetable oil and an extra oil supply during incubation had a second oil addition (1 w/v %) at day 6. In order to apply an oil addition that allows detectable population growth before nutrient depletion, the raw linseed oil suspensions with an initial concentration of 0.02% had a second oil addition at day 8 of 0.2%.

**Table 1:** The culture media tested, including the initial concentration of the carbon‐based nutrient, type of carbon‐based nutrient, concentration of the second carbon addition, number of sample sets, and incubation set

Media	Initial concentration of carbon‐based nutrient (w/v)	Type of carbon‐based nutrient	Concentration of second addition of carbon‐based nutrient (w/v)	Number of sample sets	Incubation set
1	1%	Raw linseed oil	–	2	A
2		Olive oil	–	1	A
3		Raw linseed oil	1%	2	B, C
4		Olive oil	1%	1	B
5		Stand linseed oil	1%	1	B
7		Glucose	–	1	D
8	0.2%	Glucose	–	1	D
9	0.02%	Raw linseed oil	0.2%	2	E, F
10	0%	No‐carbon	–	4	A, B, E

Note

–: not relevant.

### 2.3 Cultivation method

Each type of growth media was tested at least in one sample set, containing three replicates each, to enable analyses of the spread of the growth data. Multiple samples sets of the raw linseed oil containing media were tested in order analyze the reproducibility: Two samples sets of the initial 1% raw linseed oil suspensions that obtain a second oil addition (media 3) and two samples sets of the raw linseed oil suspensions starting with 0.02% raw linseed oil (media 9; Table 1). Minimal media without an additional carbon source were used as control media: three times a set of three shake flasks suspensions with minimal media were tested. Incubation sets, each containing sample sets of one to four types of growth media (Table 1), were tested at different time points. Suspension volumes below 80 ml were incubated in baffled shake flasks (BELLCO 500 ml) with rotary shaking at 200 rpm, aiming at a uniform distribution of media components, oxygen, and cells. Suspensions were incubated at 25°C for at least eight, up to 28 days.

### 2.4 Cell count and CFU count method

A cell count method was used to measure the total number of cells during cultivation, and a colony‐forming unit (CFU) count method was used to estimate the number of viable cells. Total cell counts were made of all suspensions. They were performed at the start of each experiment and at least four times during incubation. CFU counts were made of the 1% oil suspensions and their corresponding control suspensions without carbon at three different incubation times. CFU counts were made seven times during incubation for each type of 1% oil suspensions with a second oil feed. To obtain a biomass sample, cultivation at 200 rpm at 25°C was temporarily stopped. Each time up to 1.1 ml suspension was aseptically removed from a flask, directly after it was vigorously vortexed. A Bürker‐Türk counting chamber with a depth of 0.01 mm (Marienfeld) was used to count cells. In the case of the CFU count method, 1 ml of a decimal dilution series up to 10^−6^ was plated on agar. Malt extract agar supplemented with penicillin and streptomycin (MEA p/s) was used in duplicate for the 1% raw linseed oil CFU counts and in triplicate for 1% oil suspensions that obtained an additional oil supplement during incubation. MEA p/s was prepared according to Samson, Hoekstra, and Frisvad (2004). The CFUs on the agar plate were counted after 4–7 days of incubation at 25°C.

### 2.5 Nile red staining of cultivated cells

A fluorescent dying method, based on Beopoulos et al. (2008) and Greenspan, Mayer, and Fowler (1985), was applied to stain intracellular fatty acids in *A. melanogenum* cells cultivated in the shake flasks of sample set B and D. A 1 mg/ml stock solution of Nile red (9‐diethylamino‐5H‐benzo[alpha]phenoxazine‐5‐one, Sigma‐Aldrich) in acetone was prepared. *A. melanogenum* samples were obtained from glucose suspensions and suspensions with initially 1% raw linseed oil, 1% stand linseed oil, and 1% olive oil at least three times in the first 8 days of incubation (at days 1, 5, and 8 or at days 1, 2, 5, and 6). Cells were washed twice with 10 mmol/l ACES (Sigma) and 0.1% Tween 80, pH 6–8 and resuspended in 0.1 ml ACES‐buffer with 0.02% Tween (Van Leeuwen, Golovina, & Dijksterhuis, 2009). The Nile red solution (1 μl) was added and incubated for a minimum of 5 min at room temperature. Microscopy was performed with a Zeiss Axioplan II microscope, a Zeiss Plan NeoFluar 40×/0.75 objective, and a red 510–560 (FT 580, LP 590) filter.

### 2.6 Data analysis

Descriptive analysis was performed with GraphPad Prism version 7.02. To enable statistical analyses, concentrations were analyzed using logarithmic transformations (Agalloco & Akers, 2017). The mean log concentrations and standard deviations are calculated for each type of suspension at a single time point. These results are transformed back to median and relative standard deviations to visualize the results. Fixed effects two‐way analysis of variance (ANOVA) was used on log concentrations for data from single incubation sets to determine differences between suspensions at certain time points and differences between time points for the same suspension. Fixed effects three‐way ANOVA was used for comparisons of data from multiple incubation sets. Wald test statistics were calculated for the formulated differences, using specific contrast statements, to determine whether these contrasts were different from zero (*p* = 0.05). Effect sizes with their 95% confidence intervals were transformed to median values in the original scale of CFU and cell concentrations. ANOVA was performed with SAS version 9.4 (SAS Institute Inc., Cary, NC, USA).

## 3 RESULTS

### 3.1 Growth on 1% vegetable oil

**Figure 1:**
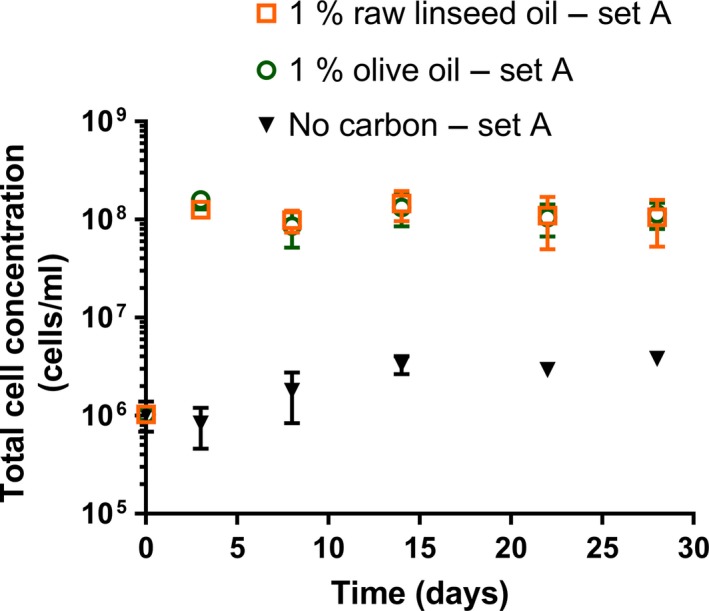
Total cell concentrations of *Aureobasidium melanogenum* (isolate CBS 140241) in cultures of liquid media with 1% raw linseed oil, 1% olive oil, or without an additional carbon‐based nutrient during shake flask cultivation (set A). The amount of vegetable oil tested was 1% (w/v). A counting chamber was used to determine the cell concentrations. Each data point represents the average of three suspensions. Error bars denote the *SD*. For some points, the error bars were shorter than the height of the symbol

**Figure 2:**
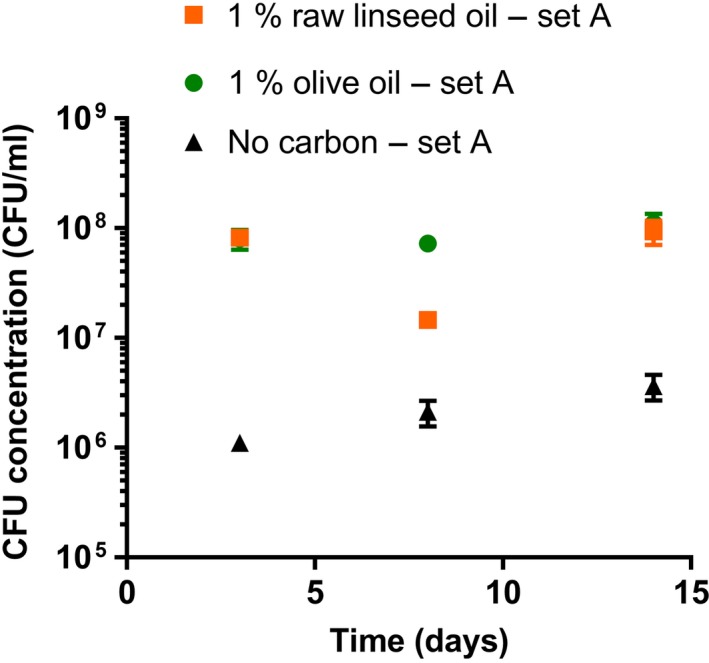
Colony‐forming unit concentrations of plated *Aureobasidium melanogenum* (isolate CBS 140241) suspensions with 1% raw linseed oil, 1% olive oil, and without a carbon source during shake flask cultivation (set A). MEA plates were used to grow the fungal colonies. Each data point represents the average of three suspensions. Error bars denote the *SD*. For some points, the error bars were shorter than the height of the symbol

The counting chamber measurements performed on the suspensions with 1% vegetable oil showed an increase in total cell concentration in the first few days of incubation (Figure 1). This could not be observed in the CFU data, because CFU concentrations in the first few days were not measured (Figure 2). The median final total cell and CFU concentrations of the different media are listed in Supporting Information Table S1.

The median final total cell concentration and the median final CFU concentration of the raw linseed oil and olive oil suspensions both differed significantly from the no‐carbon suspensions (*p* < 0.001). The median final total cell concentration of the raw linseed oil suspensions was similar compared to the median cell concentrations of the olive oil suspensions (*p* = 0.85). However, the median CFU concentration of the olive oil suspensions during incubation (Time >0 days) was significantly higher than the median CFU concentration of the raw linseed oil suspensions (*p* < 0.001). The measurement at day 8 of the raw linseed oil suspensions (Figure 2) was significantly lower compared to the concentration at days 3 and 14 (*p* < 0.001).

Although the total cell concentrations and CFU concentrations of the no‐carbon suspensions were much lower than of the oil suspensions, they did show an increase during incubation (Figures 1 and 2). For example, the median of the measurements of the no‐carbon suspensions before day 14 was significantly different from the median concentration of the measurements at day 14 and later (*p* < 0.001). Also, the median CFU concentration of the first two measurements (at day 3 and 8) and the last measurement (at day 12) of the no‐carbon suspensions of set A did show a significant difference (*p* < 0.001).

The median of the CFU concentrations measured at days 3, 8, and 14 (Figure 2) of the no‐carbon suspensions (set A) was significantly similar (*p* = 0.121) to the median of the corresponding total cell concentrations (Figure 1). In contrast, the median of the CFU concentrations of the 1% oil suspensions was significantly different compared to the median of the corresponding total cell concentrations (*p* < 0.001).

### 3.2 Growth in suspensions with an initial amount of 1% oil and second oil supply

**Figure 3:**
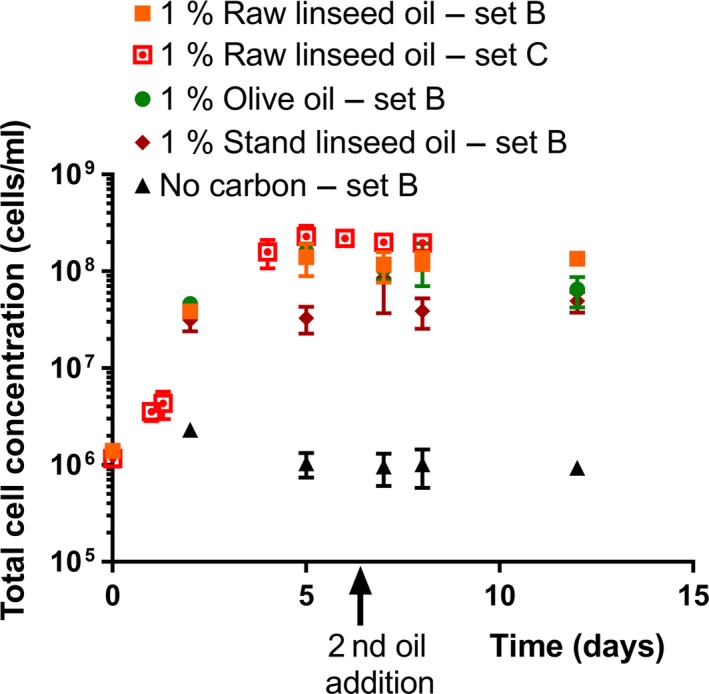
Total cell concentrations of *Aureobasidium melanogenum* (isolate CBS 140241) in liquid media with raw linseed oil, olive oil, or stand linseed oil as sole carbon source during shake flask cultivation up to 12 days. The initial amount of oil was 1% (w/v). At day 6, a second amount of oil (approx. 1% w/v) was added. A counting chamber was used to determine the cell concentrations. Each data point represents the average of three suspensions. Error bars denote the *SD*. For some points, the error bars were shorter than the height of the symbol

**Figure 4:**
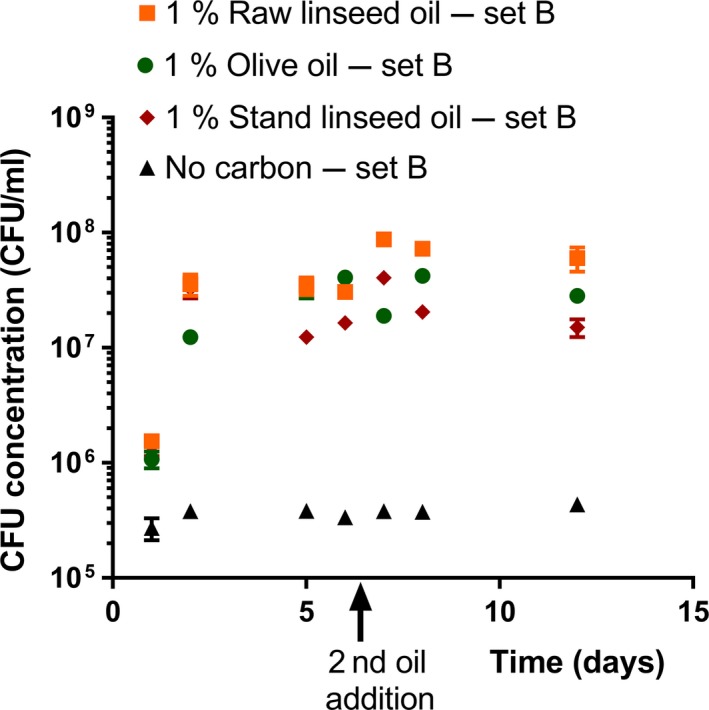
Colony‐forming unit concentrations of *Aureobasidium melanogenum* (isolate CBS 140241) in liquid media with raw linseed oil, olive oil, or stand linseed oil as sole carbon source during shake flask cultivation (set C). The initial amount of oil was 1% (w/v). At day 6, a second amount of oil (approx. 1% w/v) was added. MEA plates were used to grow the fungal colonies. Each data point represents the average of three suspensions. Error bars denote the *SD*. For some points, the error bars were shorter than the height of the symbol

The measurements performed on the suspensions with an initial amount of 1% oil and a second oil supply of 1% during incubation showed an increase in total cell and CFU concentration in the first few days of incubation (Figures 3 and 4). The median total cell and CFU concentration at day 4 and later (Time >2 days) and at day 7 and later (Time ≥7 days) of the different media are listed in Supporting Information Table S1.

The median total cell concentration of the stand linseed oil suspensions after the extra oil supply at day 6 (Time ≥7 days) was significantly higher than the median concentration just before the extra oil supply (*p* = 0.013). The median CFU concentrations of the stand linseed oil suspensions after the extra oil supply (Time ≥7 days) were also significantly higher than before (at days 5 and 6). In contrast, the median total cell concentration of the raw linseed oil and olive oil suspension (set B) measurements at day 5 and the median total cell concentration of the measurements after the extra oil supply (at days 7, 8, and 12) were significantly similar (*p* = 0.727, *p* = 0.576), while the median CFU concentrations just before and after the extra oil supply significantly differed. In the case of the olive oil suspensions, the median CFU concentration after the extra oil (2.8 × 10 CFU/ml; Supporting Information Table S1) was even lower than the median CFU concentration before the extra oil (3.2 × 10 CFU/ml, 95% confidence interval = [3.3 × 10^7^; 4.1 × 10^7^).

Each type of oil containing suspension had a significant higher median total cell and CFU concentration during incubation after day 2 (Time >2 days) and after the second oil supply (Time ≥7) than the no‐carbon suspension (*p* < 0.001). These median total cell and CFU concentrations significantly differed among the oil types (*p* < 0.05). For both time periods (Time >2 days and Time ≥7 days resp.), the raw linseed oil suspensions had the highest median total cell and CFU concentration, and stand linseed oil the lowest (Supporting Information Table S1).

The median CFU concentration at day 5 and later (Time >2 days) of the different type of oil suspensions and the no‐carbon suspensions was lower than the median total cell concentrations at day 5 and later (Supporting Information Table S1). The median of the CFU concentrations measured during incubation at days 2, 5, 7, 8, and 12 of the different suspensions was significantly different compared to the median of the corresponding total cell concentrations (*p* < 0.05).

### 3.3 Growth on 1% and 0.2% glucose

The different amounts of glucose resulted in a different yield of *A. melanogenum* cells. The counting chamber measurements performed on the glucose suspensions showed an increase in total cell concentration in the first days of incubation. The suspensions with an initial amount of 1% glucose had a final median of 1.3 × 10^8^ cells/ml (95% confidence interval = [1.2 × 10^8^, 1.5 × 10^8^]) that included the total cell concentration at days 4, 5, and 6. The measurements of the 0.2% glucose suspensions showed already a similar total cell concentrations from day 1 onwards (days 1, 1.5, 4, 5, and 6), resulting in a median final total cell concentration of 1.9 × 10^7^ cells/ml (95% confidence interval = [1.7 × 10^7^; 2.1 × 10^7^]). Results of the ANOVA test showed that these medians were significantly different (*p* < 0.001).

### 3.4 Growth in suspensions with an initial amount of 0.02% oil and second oil supply

**Figure 5:**
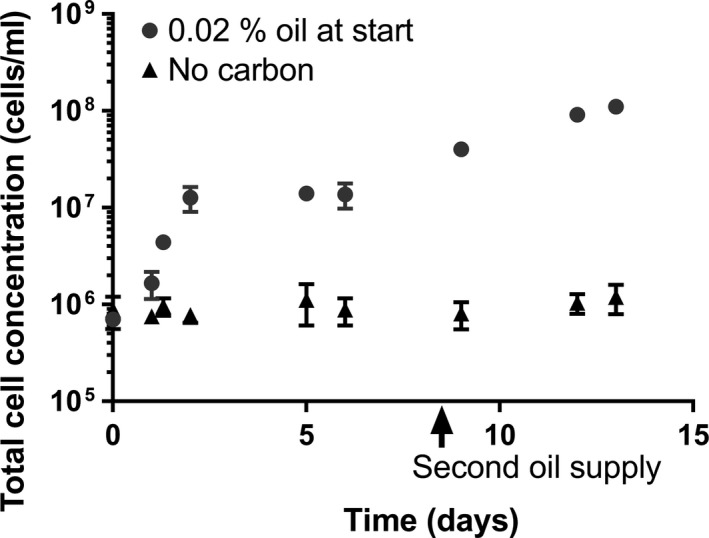
The development of *Aureobasidium melanogenum* cell concentrations in time of suspensions with 0.02% initial raw linseed oil and a second carbon source addition of 0.2 w/v % at day 8 (set E). The suspensions without carbon had no oil addition at day 8. Each data point represents the average of concentrations measured in three suspensions. Error bars denote the *SD*. For some points, the error bars were shorter than the height of the symbol

The raw linseed oil suspensions with an initial amount of 0.02% showed an increase in total cell concentration in the first days of incubation and after the second oil supply (Figure 5). In contrast, the total cell concentration of the no‐carbon suspension showed similar cell concentrations during incubation (Figure 5). The total median cell concentration of the measurements of the no‐carbon suspensions at day 0 till day 6 was significantly similar to day 9 till day 13 (*p* = 0.266).

The median total cell concentration of the measurements at days 2, 5, and 6 of set E of the 0.02% raw linseed oil suspensions, so before the extra oil addition, was significantly higher than the median concentration of the corresponding no‐carbon suspensions (*p* < 0.001, Supporting Information Table S1). After the extra oil addition to the 0.02% raw linseed oil suspensions of set E, the median total cell was significantly higher than before the extra oil addition (*p* < 0.001). Samples of the 0.02% raw linseed oil suspensions of set F also showed a significantly higher cell concentration after the extra oil addition than before (*p* < 0.001, Supporting Information Table S1).

### 3.5 Nile red staining of cultivated cells

**Figure 6:**
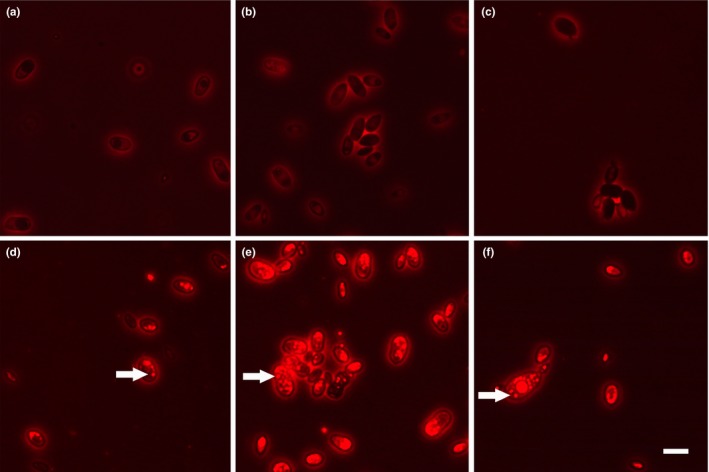
*Aureobasidium melanogenum* cells from various suspensions stained with Nile Red: (a) 0.2% glucose suspension; (b) 1% glucose suspension; (c) no‐carbon suspension; (d) 1% linseed oil suspension; (e) 1% olive oil suspension; (f) 1% stand linseed oil suspension. Red colored halos are present around cells of each type of suspension. Red dots inside cells show intracellular clusters of fatty acids (examples are marked by an arrow). Scale bar = 10 µm

Staining of the cultivated cells with Nile red revealed the presence of fatty acids inside the cells of suspensions that initially contained 1% vegetable oil (Figure 6). In suspensions with olive and raw linseed oil, red colored round shaped dots were observed at each selected time point during the first 8 days of incubation (starting at day 1). In suspensions supplemented with stand linseed oil, red dots inside the cells were recognized after 2 days of incubation. In suspensions with glucose as carbon source or without carbon intracellular, red dots were absent. Red colored halos were present around cells of each type of suspension.

## 4 DISCUSSION

### 4.1 Restrictions of the test results

In this study, a batch cultivation method was used to grow *A. melanogenum*. When using other setups, the growth conditions, for example, the amount of oxygen or distribution of cells, might be different. This can influence the growth behavior of a fungal population (Jafari, Sarrafzadeh, Alemzadeh, & Vosoughi, 2007). Therefore, conclusions that are obtained from the growth results in this paper are related to the specific cultivation method used in this study.

In this study, the final or stationary growth phase of different types of oil suspension is compared. It provides information on the use of vegetable as carbon and energy for the growth of the biofinish‐inhabiting fungus *A. melanogenum*. It also shows effects of the oil type and the amount of oil on the cell yield of *A. melanogenum*. Increasing the number of measurements performed on the oil suspensions in the first few days of incubation may allow the use of biological growth curves (Zwietering, Jongenburger, Rombouts, & Van 't Riet, 1990). This can provide a scientific backing for the data points that are included in a stationary growth phase. Also, variables, such as lag time, maximal growth rate, and moment of maximal growth, could provide additional information on the effect of the oil type and the amount of oil on the cell yield of *A. melanogenum* when cultivated with a vegetable oil as a sole carbon‐based nutrient.

### 4.2 Effect of different concentrations of oil on fungal growth

Fungal growth requires an organic carbon‐based nutrient together with several other elements such as water, nitrogen, and trace metals (Deacon, 2006; Griffin, 1994). Glucose is a commonly used carbon and energy source (Griffin, 1994). The glucose suspensions in this study showed fungal population growth and could be related to the amount of glucose used. In the tested oil suspensions, vegetable oils were the only carbon‐based nutrients present in the media. The median final concentration of the 1% glucose suspensions was similar to the 1% raw linseed oil suspensions (set A). This raises the question if the same mechanism, namely providing utilizable sources of carbon and energy, plays a role.

The suspensions without a carbon‐based nutrient showed a mean total cell concentration during incubation in the same order of magnitude as the concentration at the start of the test. No gradual increase nor decrease of the total cell concentration in time was observed for the no‐carbon suspension of set B and set E. However, looking at the results of the no‐carbon suspension set A (Figure 1), a significant increase in total cell concentration appeared in the first 14 days of incubation. The CFU concentration data of this suspension set also showed an increase in time (Figure 2). This increase could be either caused by measuring errors or it indicates that cell production has occurred. Fungal cell division or sporulation in media, where carbon‐based nutrients appear to be absent, has been mentioned in other studies (Parkinson, Wainwright, & Killham, 1989; Segers, Laarhoven, Wösten, & Dijksterhuis, 2017; Wainwright, 2005). However, it should be noted that it is unknown and, with respect to the general requirements for fungal growth, unlikely that cell production in media without carbon‐based nutrients is related to an increase in biomass of the fungal population.

The different initial oil concentrations used had an effect on the fungal growth. The oil suspensions with 1% and 0.02% oil all showed an increase in total cells concentration, followed by a stationary phase (Figures 1 and 5). A stationary phase is generally caused by the lack of an essential nutrient or the accumulation of toxic compounds (Mazzoleni et al., 2015). The median total cell concentration of the measurements performed on the oil suspensions related to the initial oil amount of 1% was at least 10^8 ^cells/ml in the stationary phase. These cell densities are within the range of yeast cell densities that can be grown under typical experimental conditions (Bath, 2008). The initial amount of 0.02% raw linseed oil clearly resulted in a lower median stationary total cell concentration than when an initial amount of 1% oil was used. It shows that the final level of total cell concentrations depends on the concentration of oil used.

The use of vegetable oils as carbon and energy source by *A. melanogenum* is likely, because the tested vegetable oils contain carbon‐based compounds which are known to be used for growth by various fungal species. For example, a carbon containing compound that is present in many vegetable oils is the long chain fatty acid named oleic acid. This acid is known to be used by several yeasts, for example, *Malassezia* spp., *S. cerevisiae*,* Candida* sp., and filamentous fungi, for example, *Aspergillus nidulans, Aspergillus niger*, and *Arthrobotrys oligospora* (Del Rio et al., 1990; Dijksterhuis, Harder, & Veenhuis, 1993; Han et al., 2002; Maggio‐Hall & Keller, 2004; Nazzaro Porro, Passi, Caprilli, Nazzaro, & Morpurgo, 1976; Radwan & Soliman, 1988; Sulter, Waterham, Goodman, & Veenhuis, 1990; Trotter et al., 2005). Also, the compound glycerol is known to provide a carbon and energy source for the growth of many fungal species, for example, *A. nidulans*,* Candida* species, *Cryptococcus curvatus*,* Fusarium oxysporum*,* Pichia membranifaciens, S. cerevisiae*, and oleaginous fungi formerly classified in the class *Zygomycetes* (Castro & Loureiro‐Dias, 1991; Chatzifragkou et al., 2011; Fabiszewska, Stolarzewicz, Zamojska, & Biaecka‐florjaczyk, 2014; Hao et al., 2015; Holst et al., 2000; Hondmann, Busink, Witteveen, & Visser, 1991; Meesters, Huijberts, & Eggink, 1996). However, not all of the carbon‐containing compounds might be easily freed from vegetable oil. For example, the use of glycerol for growth requires the full breakdown of the triglyceride or cross‐linked triglycerides that are present in oil. *Aureobasidium* isolates, including an *A. melanogenum* isolate, are known to produce extracellular lipases (Kudanga et al., 2007; Leathers, Rich, Anderson, & Manitchotpisit, 2013; Liu, Chi, Wang, & Li, 2008; Wongwatanapaiboon et al., 2016), but they might not be able to break down all types of triglycerides because lipases act chemo‐specific and region‐specific. Also, triglyceride networks of cross‐linked triglycerides might inhibit binding and activity of lipases (Shogren, Petrovic, Liu, & Erhan, 2004).

The accumulation of fatty acids in *A. melanogenum* cells of the oil containing suspensions (Figure 6) indicates the uptake of fatty acids derived from oil present in the growth medium. First of all, it is likely that *A. melanogenum* has a fatty acid uptake and transport mechanism like other well studied fungal species, for example, *A. nidulans*,* Candida neoformans*,* S. cerevisiae*, and *Y. lipolytica* (Black & DiRusso, 2003; Dulermo, Gamboa‐Meléndez, Ledesma‐Amaro, Thévenieau, & Nicaud, 2015; Kretschmer, Wang, & Kronstad, 2012). Secondly, free fatty acids are present in the surroundings of the cells. Not only a low amount of FFA is present in vegetable oils, but likely the *A. melanogenum* cells will actively increase the amount of FFA in the oil suspensions. As mentioned before, *Aureobasidium* is known to produce lipases, which may stimulate the breakdown of triglycerides in an oil containing suspension into FFA. Thirdly, the production of intracellular oil without the use of any extracellular oil from the media is unlikely. Several *Aureobasidium* strains are known to produce intracellular oil when grown in an oil‐free medium (Manitchotpisit, Price, Leathers, & Punnapayak, 2011; Wang, Li, Xin, Liu, & Chi, 2014). In the study of Wang et al. (2014), the production of intracellular lipids in an *A. melanogenum* strain is related to the amount of carbon‐based nutrient that is present in the medium. In this study, the *A. melanogenum* strain (CBS 140241) showed small intracellular lipid particles in the cells that were cultivated with oil, but it did not show intracellular fatty acid accumulation during cultivation in the oil‐free media (Figure 6). Theoretically, the presence of extracellular oil might stimulate cells to produce intracellular lipids, but it seems unlikely that *A. melanogenum* can supply itself with carbon‐based nutrients that enable growth of cells and the production of intracellular fatty acid without the use of carbon‐based nutrients that were supplied to the medium.

The demonstration of oil‐related population growth and accumulation of fatty acids in *A. melanogenum* cells indicates that the tested olive oil, raw linseed oil, and stand linseed oil provide a carbon and energy for the growth of the biofinish‐inhabiting fungus in shake flak cultivation. The finding of oil‐related *A. melanogenum* growth when using raw linseed or olive oil as carbon‐based nutrient is in line with the findings of Peeters et al. (2018). Although the methods to demonstrate growth differed, it indicates that various types of raw linseed oil or olive oil are suitable sources of carbon and energy for *A. melanogenum*. To our knowledge, stand linseed oil has not been tested as carbon and energy source for *A. melanogenum* before. Therefore, the conclusion that stand linseed oil provides carbon and energy for *A. melanogenum* is restricted to the specific stand linseed oil that was used in this study.

### 4.3 Differences in total cell versus CFU concentration

The median CFU concentrations of the oil suspensions were lower than the corresponding total cell concentrations. This difference can be attributed to the fact that total cell counts include dead cells and viable cells, while CFU counts only reveal the colony‐forming ability of viable cells. In addition, the CFU counts are an estimate of the viable cells, because some CFU will represent a budding cell or agglomerated singles cells and not a well separated single cells (Sutton, 2012). Manual CFU counts and total cell counts are both known for their high inherent error (Prescott, 1975; Sutton, 2012). These methods are subjected to variety of random error including, sampling, dilution, and technician counting errors (Prescott, 1975; Sutton, 2012). It explains why the *SD* of the measured total cell and CFU concentrations at a single time point were frequently in the same order of magnitude as the median cell concentration.

More distinct fluctuations were observed in the CFU concentrations along the time line of the incubated oil suspensions than in the corresponding total cell concentrations (Figures 1–2). For example, the raw linseed oil suspension of set A showed a drop of CFU concentration at day 8, which significantly differed from the other data points (Figure 2). In addition, the stand linseed oil suspension showed a lower median CFU concentration at day 5 than at day 2 (Figure 4), while the corresponding total cell counts had a similar concentration at these two time points (Figure 3). These fluctuations in CFU concentration can be attributed to an actual fluctuation in viable cells, but are more likely related to the high measuring error of this method.

### 4.4 Effect of second oil addition on fungal growth

Not all second oil additions showed an effect on the *A. melanogenum* cell yield. In the case of the suspension with initially 1% olive and raw linseed oil and a second oil supply, a second oil increase in the total cell concentration was absent. This suggests that the oil present in the 1% oil suspensions is not a limiting growth factor. The significant increase after the second oil addition in CFU concentration of the initially 1% raw linseed oil suspensions lowers the credibility of this assumption, but the significant decrease in CFU concentration of the olive oil suspensions after the second oil supply indicates that the CFU method might not be very accurate.

In contrast to the suspension with initially 1% olive and raw linseed oil and a second oil supply, two stationary phases were observed in the total cell and CFU data of the stand linseed oil suspensions. The first stationary phase is assumed to be caused by a shortage of a consumable carbon and energy source, as both the cell and CFU concentration showed a further increase after the second oil supply. Probably the second stationary phase is caused by a limiting growth factor, other than the amount of oil available.

In the case of the 0.02% raw linseed oil suspensions, a second increase in total cell concentration occurred after a second oil addition. This suggests that the first stationary phase is caused by a limitation in the amount of raw linseed oil available. It is unclear if the second stationary phase (final total cell concentration) is induced by the lack of raw linseed oil, by the lack of another essential nutrient or the accumulation of toxic compounds.

### 4.5 The effect of different carbon‐based nutrients on the cell yield of *Aureobasidium melanogenum*

A clear effect between the use of olive and raw linseed oil on the total cell yield in 1% oil suspensions is absent. The use of 1% raw linseed oil and olive oil as sole carbon‐based nutrient revealed similar final total cell concentrations. This conclusion is based on the results of the 1% raw linseed suspensions of set A and of the 1% oil suspensions with a second oil addition of set B (Figures 1 and 3). It is unclear if the use of 1% raw linseed oil and olive oil as sole carbon‐based nutrient revealed similar final viable cell concentration. The median CFU concentration of all data points measured in the raw linseed oil suspensions with 1% oil at the start of the test was significantly different compared with the olive oil suspensions. However, this measured difference might be explained by the inaccuracy of the selected method. In the 1% oil suspensions (media 1, 2), the median CFU concentration of the raw linseed oil suspensions is significantly lower than the olive oil suspensions due to a drop in CFU concentration at day 8. In contrast, the initially 1% oil suspensions with a second oil addition show a higher median CFU concentration of measurements performed before the second oil addition for the raw linseed oil suspensions than for the olive oil suspensions. Again, the difference is due to a drop in CFU concentration at a single time point (day 6).

The absence of a clear difference in cell growth between the use of olive and raw linseed oil in 1% oil suspensions might be explained by the absence of information on the initial growth behavior and the high amount of oil in the tested media. At first, the absence of a clear difference in the total cell yield of the 1% olive and raw linseed oil suspensions is surprising. For one thing, fungi are known to grow differently due to the use of different carbon sources (Sati & Bisht, 2006; Yang, Ke, & Kuo, 2000). In addition, lipases in the *A. melanogenum* suspensions are suspected to free different amounts of potentially utilizable fatty acids and glycerol compounds when using different oil types, because substrate‐specific activities of fungal lipases are common (Avelar et al., 2013; Dheeman, Antony‐Babu, Frías, & Henehan, 2011; Song, Qi, Hao, & Qu, 2008). However, comparing the final total cell concentration (stationary phase) of suspensions that are cultivated in conditions where the limiting growth factor is something other than the carbon‐based nutrient is probably not the best method to find differences between oil types. Parameters that can be obtained from the initial growth phase when sufficient data points are present, such as maximal growth rate, are more likely to be different in this type of growth conditions (Naher, Radziah, Halimi, Shamsuddin, & Razi, 2008).

The total cell and CFU results indicated that in the case of an equal initial oil concentration, stand linseed oil provides less carbon and energy for *A. melanogenum* growth than olive and raw linseed oil. The stand linseed oil suspensions revealed a lower median final total cell and CFU concentration than the raw linseed and olive oil suspensions of set B (Figures 3 and 4, Supporting Information Table S1). This could be explained by the degree of cross‐linked molecules. Stand linseed oil has a higher degree of cross‐linked triglycerides than raw linseed and olive oil (Zovi et al., 2011), and cross‐linking can influence the growth of *A. melanogenum* on oil (Peeters et al., 2018). The lipases produced by *A. melanogenum* might be unable to act on big molecules of cross‐linked triglycerides. Other enzymes not produced by *A. melanogenum* are probably needed as well to breakdown the big molecules present in stand linseed.

## CONFLICT OF INTEREST

The authors declare no conflict of interest.

## AUTHORS CONTRIBUTION

EN and SR carried out the experiments. EN wrote the manuscript with support from EH, MS, SR, RS, and OA. EH performed the statistical analysis. EN and EH analyzed the data. EN and MS conceived the original idea. RS and OA supervised the project.

## ETHICS STATEMENT

None required.

## Data Availability

The data will be available on request from the corresponding author.
