# Supplementary material for: Vegetable oils as carbon and energy source for Aureobasidium melanogenum in batch cultivation
Source: Microbiologyopen. 2018 Dec 4;8(6):e00764. doi: 10.1002/mbo3.764 (PMC6562153; doi:10.1002/mbo3.764)
Supplement: Supplementary file 1 [file MBO3-8-e00764-s001.docx]

Table S1: Median total cell and CFU concentrations of measurements performed on oil containing media and no-carbon suspensions. CI = confidence interval, n.a. = not available.

| Carbon-based nutrient  & incubation set  (Media type) | Measurements included in median concentration (day) | Median total cell concentration (cells ml^-1^) | 95 % CI lower | 95 % CI upper | Median CFU concentration (CFU ml^-1^) | 95 % CI lower | 95 % CI upper |
| --- | --- | --- | --- | --- | --- | --- | --- |
| No-carbon, set A  (media 10) | > 0 | 2.2E+06 | 1.8E+06 | 2.6E+06 | 2.0E+06 | 1.7E+06 | 2.3E+06 |
| Raw linseed oil, set A, (media 1) | > 0 | 1.1E+08 | 9.3E+07 | 1.3E+08 | 4.8E+07 | 4.1E+07 | 5.5E+07 |
| Olive oil, set A  (media 2) | > 0 | 1.1E+08 | 9.5E+07 | 1.3E+08 | 8.5E+07 | 7.4E+07 | 9.9E+07 |
| No-carbon, set B (media 10) | > 2 | 9.5E+05 | 8.1E+05 | 1.1E+06 | 3.8E+05 | 3.6E+05 | 4.1E+05 |
| Raw linseed oil, set B (media 3) | > 2 | 1.3E+08 | 1.1E+08 | 1.5E+08 | 5.2E+07 | 4.9E+07 | 5.6E+07 |
| Olive oil, set B  (media 4) | > 2 | 1.1E+08 | 9.0E+07 | 1.3E+08 | 3.1E+07 | 2.9E+07 | 3.4E+07 |
| Stand linseed oil, set B (media 5) | > 2 | 4.6E+07 | 3.9E+07 | 5.4E+07 | 1.9E+07 | 1.8E+07 | 2.0E+07 |
| Raw linseed oil, set C (media 3) | > 2 | 2.0E+08 | 1.7E+08 | 2.3E+08 | n.a. | n.a. | n.a. |
| No-carbon, set B (media 10) | ≥ 7 | 9.4E+05 | 7.8E+05 | 1.1E+06 | 4.0E+05 | 3.6E+05 | 4.3E+05 |
| Raw linseed oil, set B (media 3) | ≥ 7 | 1.3E+08 | 1.0E+08 | 1.5E+08 | 7.2E+07 | 6.6E+07 | 7.9E+07 |
| Olive oil, set B  (media 4) | ≥ 7 | 9.3E+07 | 7.7E+07 | 1.1E+08 | 2.8E+07 | 2.6E+07 | 3.1E+07 |
| Stand linseed oil, set B (media 5) | ≥ 7 | 5.2E+07 | 4.3E+07 | 6.3E+07 | 2.3E+07 | 2.1E+07 | 2.5E+07 |
| Raw linseed oil, set C (media 3) | ≥ 7 | 2.0E+08 | 1.6E+08 | 2.5E+08 | n.a. | n.a. | n.a. |
| 10 (No-carbon), set E (media 10) | 2, 5, 6 | 8.8E+05 | 7.4E+05 | 1.1E+06 | n.a. | n.a. | n.a. |
| Raw linseed oil, set E (media 9) | 2, 5, 6 | 1.3E+07 | 1.1E+07 | 1.6E+07 | n.a. | n.a. | n.a. |
| Raw linseed oil, set F (media 9) | 5, 6, 6.3, 8 | 1.1E+07 | 9.4E+06 | 1.4E+07 | n.a. | n.a. | n.a. |
| Raw linseed oil, set E (media 9) | 9, 12, 13 | 7.4E+07 | 6.2E+07 | 8.8E+07 | n.a. | n.a. | n.a. |
| Raw linseed oil, set F (media 9) | 11, 12, 14 | 7.5E+07 | 5.9E+07 | 9.4E+07 | n.a. | n.a. | n.a. |
